# Late-onset major depression and subthreshold depressive symptoms as early clinical correlates of brain small vessel disease

**DOI:** 10.1093/ijnp/pyag024

**Published:** 2026-05-08

**Authors:** Calypso A Mitkani, Thomas J Tegos, Vasilios K Kimiskidis, Stavroula Koukou, Konstantinos N Fountoulakis

**Affiliations:** Department of Neurology, Agios Pavlos General Hospital of Thessaloniki, Thessaloniki, Greece; Department of Psychiatry, School of Medicine, Faculty of Health Sciences, Aristotle University of Thessaloniki, Thessaloniki, Greece; WHO Collaborating Center on Quality in Mental Health, School of Medicine, Faculty of Health Sciences, Aristotle University of Thessaloniki, Thessaloniki, Greece; Department of Neurology, School of Medicine, Faculty of Health Sciences, Aristotle University of Thessaloniki, Thessaloniki, Greece; Department of Neurology, School of Medicine, Faculty of Health Sciences, Aristotle University of Thessaloniki, Thessaloniki, Greece; Department of Neurology, Agios Pavlos General Hospital of Thessaloniki, Thessaloniki, Greece; Department of Psychiatry, School of Medicine, Faculty of Health Sciences, Aristotle University of Thessaloniki, Thessaloniki, Greece; WHO Collaborating Center on Quality in Mental Health, School of Medicine, Faculty of Health Sciences, Aristotle University of Thessaloniki, Thessaloniki, Greece

**Keywords:** depression, stroke, small vessel disease

## Abstract

Late-onset depression (LOD) and subthreshold depressive symptoms (SDS) have been associated with cerebral small vessel disease (SVD), but the evidence regarding pre-stroke depression remains limited. This study examined the frequency of DSM-5 LOD and SDS prior to the first non-embolic lacunar stroke. Fifty-eight patients (mean age 62.2 ± 8.2 years; 41.4% female) with a first lacunar ischemic stroke underwent Magnetic Resonance Imaging (MRI) (T1, T2, FLAIR sequences) and psychiatric evaluation using the MINI, with data from caregivers and medical records. Patients were classified as having LOD (D), SDS (S), combined (S/D), or none (nD/S). Statistical analyses included Mann–Whitney *U* and Fisher’s Exact tests. Of the total sample, 63.8% (*n* = 37) exhibited LOD or SDS, with reported onset on average 5-7 years prior to stroke (mean onset age: 55.5 years). Females exhibited higher prevalence of depressive symptoms (87.5% vs 47.1%, *P* = .001). Hypertension (75.7%) and dyslipidemia (62.2%) were the most common vascular risk factors. No patient had manic or hypomanic episodes. Premorbid depressive symptoms were common and may precede the clinical manifestation of cerebrovascular disease Given the retrospective and observational design, these findings should be regarded as hypothesis-generating rather than causal. Prospective longitudinal studies incorporating quantitative SVD imaging markers are required.

## Introduction

Lacunar cerebral infarcts are small (<5-20 mm) ischemic strokes caused by occlusion of deep penetrating arteries.^1^ They represent approximately 20%-25% of all ischemic strokes.^2^ These strokes are often associated with hypertension, diabetes mellitus, and dyslipidemia.^1^^,^^2^

Depression is a common psychiatric disorder with a current annual prevalence around 8%,^3^ higher than older estimates of 4.5%.^4^ Lifetime prevalence is about 20%^5^^,^^6^ Studies have linked late-onset depression (LOD) with vascular pathology, with depression often appearing years before overt cerebrovascular disease.^7^^,^^8^ The vascular depression hypothesis suggests that SVD, particularly white matter hyperintensities, may disrupt fronto-subcortical circuits, leading to mood disorders; however, the specific contribution of lacunar infarcts per se remains less clearly established.^9^

Depression is also a known risk factor for stroke.^10^ Therefore, the presence of LOD or SDS may reflect an underlying vascular vulnerability in advance of clinically overt stroke. The present study aims to examine the frequency, clinical characteristics, and temporal patterns of Diagnostic and Statistical Manual of Mental Disorders, Fifth Edition (DSM-5) LOD and subthreshold depressive symptoms (SDS) prior to the first non-embolic lacunar stroke, as well as their associations with demographic and vascular risk factors.

## Materials and methods

### Patient selection

Fifty-eight patients with first lacunar ischemic stroke were included. Patients were recruited from the Department of Neurology, Agios Pavlos General Hospital of Thessaloniki, Greece, and AHEPA University Hospital of Thessaloniki, Greece, over the study period 2021-2023. The study followed a retrospective observational design. The mean age was 62.2 ± 8.2 years. Inclusion criteria: (1) hospital admission for first acute ischemic lacunar stroke confirmed by MRI; (2) radiological evidence of small vessel disease (SVD), operationally defined as the presence of lacunes and/or white matter hyperintensities (WMH) on FLAIR sequences, in line with established neuroimaging standards for SVD^1^ However, a formal grading system (eg, STRIVE/STRIVE-2 criteria or Fazekas scale) was not systematically applied due to the retrospective nature of the study. Microbleeds and enlarged perivascular spaces were not systematically assessed due to limitations of the routine clinical MRI protocol**.** Exclusion criteria: atrial fibrillation, severe carotid stenosis, or any cardioembolic source, which were ruled out by ECG, duplex ultrasound, and echocardiography. Vascular risk factors (including hypertension, diabetes mellitus, dyslipidemia, smoking, and alcohol use) were obtained from medical records, patient history, and medication use at the time of admission.

The study was conducted in accordance with the Declaration of Helsinki and was approved by the local institutional review board. All participants provided written informed consent.

### MRI imaging

All patients underwent 1,5 T MRI with axial T1-weighted, T2-weighted, diffusion-weighted imaging (DWI), and FLAIR sequences. Lacunar stroke was defined as a subcortical infarct <15 mm in diameter in the territory of a single perforating arteriole^1^ Imaging was reviewed by experienced clinicians as part of routine clinical practice and was not blinded to clinical data. MRI assessment was qualitative; quantitative measures of SVD burden (eg, Fazekas score or lacune count) were not performed.

### Psychiatric assessment

A psychiatrist conducted MINI interviews and reviewed medical/psychiatric histories with caregiver input. LOD was defined as first major depressive episode ≥50 years, while SDS referred to clinically relevant depressive symptoms lasting ≥2 weeks, not meeting full DSM-5 MDD criteria, but associated with subjective distress and functional impairment. Given the retrospective nature of symptom assessment, potential recall bias and informant-related inaccuracies cannot be excluded.

### Statistical analysis

Descriptive statistics included means, standard deviations, and frequencies. Continuous variables were assessed for distributional properties. Due to small sample size and group imbalances, non-parametric tests (Mann–Whitney *U*) were primarily used for pairwise comparisons, and Fisher’s Exact test was used for categorical variables when expected cell frequencies were <5.

In addition, exploratory parametric analyses (including Analysis of Variance [ANOVA] and multivariate analysis of variance [MANOVA]) were performed to examine group differences across multiple variables, given their relevance for hypothesis generation. These analyses were interpreted cautiously.

Significance was set at *P* <.05. Given the exploratory nature of the study, no correction for multiple comparisons was applied.

## Results

### Sample characteristics

Participants were classified into 4 groups: (1) LOD (D), (2) SDS (S), (3) no depression (nDS), and (4) combined depression group (S/D).

The combined depression group (S/D) accounted for 63.79% (*N* = 37) of the total sample. A significantly higher proportion of females (*N* = 21, 87.5%) than males (*N* = 16, 47.06%) belonged to this group (Fisher’s Exact test, *P* = .001).

There was no documented history of manic, mixed, or hypomanic episodes in any patient. Depressive symptomatology was chronic in all cases, without symptom-free intervals, and was reported as relatively refractory to treatment based on retrospective clinical information.

Descriptive statistics for categorical variables are presented in Table 1.

**Table 1:** Frequencies of vascular and clinical risk factors across patient subgroups and comparison using Fisher’s Exact test.

**Subgroup**	**nDS**		**S**		**D**		**D/S**		**All Grps**	***P***
	***N***	**%**	***N***	**%**	***N***	**%**	***N***	**%**	***N***	
**Diabetes melitus**	10	47.62	6	40.00	8	36.36	14	37.84	24	.830
**Hypertention**	16	76.19	11	73.33	17	77.27	28	75.68	44	.500
**Coronary disease**	4	19.05	1	6.67	5	22.73	6	16.22	10	.853
**Hypothyroidism**	2	9.52	3	20.00	4	18.18	7	18.92	9	.428
**Dyslipidemia**	12	57.14	9	60.00	14	63.64	23	62.16	35	.571
**Alcoholism**	1	4.76	3	20.00	2	9.09	5	13.51	6	.427
**Smoking**	12	57.14	6	40.00	9	40.91	15	40.54	27	.914
**Substance use**	0	0.00	0	0.00	2	9.09	2	5.41	2	.635
**Alcohol**	1	4.76	1	6.67	1	4.55	2	5.41	3	.781
**Family history of any mental disorder**	7	33.33	12	80.00	9	40.91	21	56.76	28	.186
**Family history of dementia**	3	14.29	3	20.00	4	18.18	7	18.92	10	.688

All categorical comparisons were performed using Fisher’s Exact test due to small expected cell counts.

### Primary analyses

Primary analyses focused on comparisons between diagnostic groups (D, S, and nDS).

The D group included 50% of females (*N* = 12) and 29.41% (*N* = 10) of males; this difference did not reach statistical significance (Fisher’s Exact test, *P* = .111). The S group included 37.50% of females (*N* = 9) and 17.65% of males (*N* = 6), with no statistically significant difference between sexes (Fisher’s Exact test, *P* = .089).

The mean age at onset in the D group was 57.59 ± 9.75 years, with a mean duration of 7.31 ± 4.05 years. In the S group, the mean age at onset was 52.40 ± 7.91 years, and the mean duration was 5.26 ± 3.93 years. No statistically significant differences were observed between the 2 groups (Table 2).

**Table 2:** Means and standard deviations of continuous clinical and demographic variables across patient subgroups.

**Subgroup**	**Sex**	**N**	**Age**		**Education**		**Paternal age**		**Maternal age**		**First episode age at onset**		**Duration since first episode**	
			**Mean**	**SD**	**Mean**	**SD**	**Mean**	**SD**	**Mean**	**SD**				
**nD/S**	**Females**	3	62.33	2.52	2.67	0.58	26.00	4.58	23.33	4.73				
	**Males**	18	62.67	6.85	2.83	1.04	26.72	3.94	24.00	3.93				
**S**	**Females**	9	57.00	11.22	3.00	1.00	32.67	6.40	26.78	6.98	52.11	10.03	4.89	3.18
	**Males**	6	58.67	3.44	2.50	0.84	34.17	9.33	29.17	5.60	52.83	3.76	5.83	5.15
**D**	**Females**	12	67.08	8.90	2.25	0.97	31.50	8.03	25.92	6.69	60.08	8.26	7.00	3.64
	**Males**	10	62.30	7.02	3.10	0.57	33.00	5.68	26.90	4.04	54.60	10.97	7.70	4.67
**All Grps**		58	62.21	8.17	2.74	0.93	30.45	6.73	25.83	5.39	55.48	9.30	6.48	4.08

Between-group comparisons were performed using non-parametric tests due to small sample sizes. Standardized effect sizes (Hedges’ g) are provided for key comparisons. All analyses are exploratory.

Descriptive statistics for continuous variables are presented in Table 2.

### Subgroup and exploratory analyses

Subgroup analyses (including sex-specific comparisons, family history, and vascular risk factors), as well as multivariate analyses, were conducted on an exploratory basis and should be interpreted with caution.

Multivariate analysis of variance examining differences among the 3 groups (D, S, and nDS) in age, paternal and maternal age, and education revealed a significant overall effect (Wilks’ Lambda = 0.678, F = 2.785, *P* = .008). Given the limited sample size and unequal group distributions, this analysis was considered exploratory.

Post-hoc Scheffe tests indicated significant differences in age between D and S groups (57.66 ± 8.77 vs. 64.91 ± 8.28; *P* = .03), as well as in paternal age between nDS and D (26.62 ± 6.73 vs. 32.18 ± 6.93; *P* = .02) and between nDS and S (26.62 ± 6.73 vs 33.26 ± 7.42; *P* = .009). Standardized effect sizes suggested moderate-to-large effects (Hedges’ g ranging from −0.80 to −0.92). These findings emerged from exploratory analyses and were interpreted cautiously.

Family history of mental disorder was reported in 40.91% of the D group compared to 52.78% of the remaining sample, and in 75% of the S/D group compared to 53.33% of the remainder. A family history of dementia was present in 16.67% of patients with depression and 18.18% of those without. None of these differences reached statistical significance (Table 1). Similarly, no significant differences were observed when comparing patients with versus without SDS.

The most common vascular risk factors in the S/D group were hypertension (75.68%) and dyslipidemia (62.16%). No statistically significant differences were observed between sexes or between diagnostic groups and the remainder of the sample (Table 1).

## Discussion

The results of this study in 58 patients with first-ever lacunar stroke indicate that almost two-thirds (63.8%) reported LOD or SDS, with symptom onset in their mid-fifties, approximately 5-7 years before the cerebrovascular event. Female patients were more likely to experience depressive symptoms of any type, a finding consistent with established epidemiological data on late-life mood disorders**.** Although some between-group comparisons did not reach statistical significance, descriptive trends suggested that subthreshold symptoms may develop earlier and may be associated with cerebrovascular events at a younger age; however, these observations should be interpreted cautiously. The observation that paternal age was associated with depressive symptom groups in the present sample is noteworthy; however, this finding should be interpreted cautiously and considered exploratory**,** given the limited sample size. Advanced paternal age has been associated with several neuropsychiatric conditions, including psychotic disorders, although**.** Its relevance in the present context remains speculative. In the literature, most studies have focused on post-stroke depression, with prevalence estimates of approximately 30%-35%, while only indirect associations with pre-stroke depression have been explored. Causality remains unclear.^11^ Accordingly, the present findings should not be interpreted as evidence of a causal relationship. Our findings suggest that depressive symptoms, particularly in women, may be reported before the clinical manifestation of stroke, and could be associated with underlying cerebrovascular processes. Similar preliminary results were reported by our group.^12^

This study focused on lacunar strokes, which constitute a distinct subset of ischemic strokes, representing about one quarter of all ischemic events.^2^ Small vessel disease—particularly white matter hyperintensities and gray matter changes— has been associated with disruptions in frontolimbic circuits, implicated in LLD.^9^ However, as quantitative measures of SVD burden were not available in the present study, direct mechanistic inferences cannot be drawn. While most evidence has focused on white matter hyperintensities, the contribution of lacunar infarcts themselves remains less clearly established. Progression of vascular burden, as evidenced by MRI white matter abnormalities and brain atrophy, has been associated with increased rates of depressive symptoms.^13^

Overall, these findings are broadly consistent with, but do not prove, the vascular depression hypothesis,^7^ which proposes that cerebrovascular pathology contributes to the onset of depression in later life. Female sex appears to be a consistent associated factor, as women have higher rates of both major depressive disorder and subsyndromal depression.^14^ The association between paternal age and depression, previously observed by our group,^12^ should be regarded as hypothesis-generating rather than confirmatory, and may reflect unmeasured familial or developmental factors and underlying genetic or epigenetic vulnerabilities.^15^ There is also substantial literature indicating that depression is associated with an increased risk of stroke, suggesting a potentially bidirectional relationship.^10^ However, the current study lacks a control group and is based on a relatively small sample, which limits the generalizability and precludes causal inferences.

In conclusion, our findings suggest that late-onset depressive symptoms may precede and be associated with an underlying burden of SVD in some patients. Although these results are compatible with the vascular depression hypothesis, they do not justify specific management changes beyond standard vascular risk control. Larger, prospective studies are required to clarify the directionality of this relationship and to evaluate whether depression could serve as an early clinical marker of SVD and its potential predictive value for stroke or cognitive impairment.

## Data Availability

The data that support the findings of this study are available from the corresponding author upon reasonable request.
